# PD50 - Impaired lung function in asthmatic schoolchildren – evaluation of risk factors

**DOI:** 10.1186/2045-7022-4-S1-P50

**Published:** 2014-02-28

**Authors:** Hanna Knihtilä, Anne Kotaniemi-Syrjänen, Pekka Malmberg, Anna Pelkonen, Mika Mäkelä

**Affiliations:** 1 HUCH Skin and Allergy Hospital, Helsinki, Finland

## 

In this retrospective study we investigated risk factors for impaired lung function based on medical records of asthmatic children treated in a tertiary hospital between October 2004 and December 2008. Baseline characteristics, lung function measurements and current medication were charted. Those 430 children with data on ≥2 lung function measurements performed ≥5 years apart, were included in the analyses.

**Figure 1:**
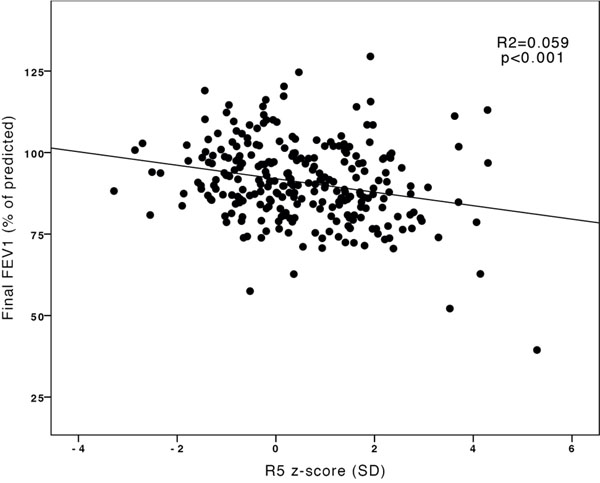


Altogether 91 (21%) children had abnormal findings in the first lung function measurement (n=38/265 in oscillometry performed at the median age of 4.2 years, and n=53/165 in spirometry performed at the median age of 7.6 years) and 183 (43%) in the last spirometry performed at the median age of 13.8 years. There were significant correlations between the oscillometry and last spirometry parameters: r=-0.243 between R5 z-score and FEV1 (p<0.001)(Fig.1), and r=-0.197 between R5 z-score and FEV1/FVC (p=0.001). Correlations between the first and last spirometry parameters were also significant: r=0.547 for FVCs, r=0.486 and for FEV1s, r=0.450 for FEV1/FVCs, and r=0.496 for ln(MEF50%)s (p<0.001 in every comparison). There was a significant, albeit mild, correlation between the need of medication and decrease in FEV1/FVC and MEF50% (rs=-0.098; p=0.043, and rs=-0.097; p=0.045). In multivariate analyses, male gender, a birth weight of <1500 g, duration of asthma, and abnormal oscillometry or spirometry parameters in the first lung function measurement were significant risk factors for impaired lung function in adolescence.

In conclusion, male gender, a very low birth weight, and the duration of asthma interfere with the lung function development. In addition, impaired lung function appears to persist despite regular asthma control medication.

